# Exploring the role of Life Essential 8 in patients diagnosed with systemic lupus erythematosus and rheumatoid arthritis

**DOI:** 10.1007/s10067-024-07114-z

**Published:** 2024-08-21

**Authors:** Xiaoying Wang, Yintao Bai, Xiaolan Zou, Xincheng He, Xiaoxiang Xie, Jinli Ru

**Affiliations:** 1 Second Clinical Medical College of Shanxi Medical University, Taiyuan, 030000 China; 2 Department of General Medicine, Second Hospital of Shanxi Medical University, Taiyuan, 030000 China

**Keywords:** Arthritis, Cardiovascular risk factors, Clinical disease activity, Life Essential 8, Lupus erythematosus, Rheumatoid

## Abstract

**Objective:**

To investigate the distribution of the Life Essential 8 (LE8) score among adult patients with systemic lupus erythematosus (SLE) and rheumatoid arthritis (RA) and explore its association with disease activity.

**Methods:**

A cross-sectional study was conducted to select adult patients with SLE and RA who were treated in the general department of the Second Hospital of Shanxi Medical University between May 2022 and September 2023. Through questionnaires, patients’ diet, sleep, smoking habits, and daily exercise were evaluated. Additionally, blood glucose, blood lipids, inflammatory markers, and other relevant data were collected to assess the LE8 levels of the participants. The data was analyzed using SPSS 26.0. Both univariate and ordered multivariate logistic regression were employed to explore the distribution and influencing factors of the LE8 score among the patients.

**Results:**

A total of 43 adult cases of SLE and 55 RA cases were studied, encompassing 11 males and 87 females with a mean age of 49.12 ± 15.86 years. The LE8 score averaged at 68.82 ± 12.29; specifically, the LE8 behavior score was 60.91 ± 17.78, and the LE8 factor score was 77.05 ± 14.30. The disease activity scores of both conditions showed a negative correlation with LE8. As DAS28 (*r* =  − 0.96, *P* < 0.05) and SLEDAI (*r* =  − 0.807, *P* < 0.05) scores increased, the LE8 score decreased. A low SLEDAI score serves as a protective factor for LE8 (OR (95% CI) = 0.07 (0.01, 0.37), *P* = 0.02). Furthermore, among patients with RA (OR (95% CI) = 0.03 (0.00, 0.22), *P* = 0.001) and SLE (OR (95% CI) = 0.06 (0.01, 0.35), *P* = 0.002), individuals boasting higher LE8 scores exhibit a reduced 10-year cardiovascular risk.

**Conclusions:**

Patients suffering from RA and SLE often exhibit low LE8 scores, reflecting a concerning cardiovascular health status—particularly in cases of high disease activity. Hence, it is imperative to prioritize the cardiovascular well-being of rheumatic patients.

**Table:** 

**Key Points** *• Research has revealed that individuals suffering from RA and SLE exhibit lower LE8 scores, potentially attributed to alterations in disease activity* *• In this study, no statistically significant associations were discerned between inflammatory markers and LE8 scores among patients with RA and SLE. Nevertheless, among SLE patients specifically, a notable correlation was observed between ds-DNA levels and LE8 factor scores.* *• Enhancing the compliance rate for the LE8 target among patients with RA and SLE could potentially mitigate the cardiovascular risk associated with these conditions.*

**Supplementary Information:**

The online version contains supplementary material available at 10.1007/s10067-024-07114-z.

## Introduction

Rheumatism is a large class of heterogeneous diseases with joints, bones, and muscles as the main symptoms, which can involve internal organs. Patients with ARDs (autoimmune rheumatic diseases, ARDs) are often accompanied by varying degrees of inflammatory activities, resulting in lipid metabolism disorders, insulin disorders, production of anti-lipoprotein antibodies, and a series of changes, thus promoting the occurrence of atherosclerosis. Chronic inflammation is an important reason for the increased risk of cardiovascular disease in patients with ARDs [1]. Patients with ARDs have a higher cardiovascular risk. Compared with ordinary people, the risk of cardiovascular disease in ARDs is 68% higher on average, and the age of developing cardiovascular disease is earlier [2, 3]. In order to improve the level of cardiovascular health and reduce the mortality of cardiovascular and cerebrovascular diseases/stroke in 2010, the USA proposed seven ideal cardiovascular health behaviors and factors (ideal cardiovascular health, ICH) [4]. In August 2022, the definition of ICH was updated to include sleep health, renamed LE8 (Life Essential 8, LE8), including nicotine exposure, sleep health, physical activity, diet, body mass index, non-high-density lipoprotein, blood pressure, fasting blood glucose, and glycosylated hemoglobin. After the introduction of the LE8 concept, many researchers studied the distribution of LE8. Studies have shown that the satisfaction level of ideal LE8 status among residents both domestically and internationally is not optimal [5, 6]. Hernandez-Martinez et al. [7] found that the distribution of ICH in patients with SLE (systemic lupus erythematosus, SLE) was not optimistic, and only 30.3% SLE patients met 5–7 items of ideal cardiovascular health in the study. Wang et al. [8] found that the ICH score was related to the risk of RA, and the risk of RA increased with the decrease of the ICH score. After the LE8 definition was updated in 2020, a study conducted in the USA revealed that, among the 1424 RA patients examined, individuals with medium and low LE8 scores exhibited an increased all-cause mortality risk of 85.8% and 129.5% compared to those with high LE8 scores, respectively. Furthermore, for each additional point increase in the LE8 score, the all-cause mortality risk reduces by 2.6% [9]. This suggests the significant value of LE8 in reducing cardiovascular risks and promoting cardiovascular health among patients with rheumatic diseases.

This study centers on adult SLE and RA patients who visited the general practice clinic and inpatient department of the Second Hospital of Shanxi Medical University. We amassed general data about the participants, such as age, income, and educational attainment, evaluated their disease activity and LE8 scores, and recorded inflammatory markers. The aim of this study is to delve into the distribution of LE8 among this cohort, scrutinize the correlation between LE8 and various factors like disease activity and laboratory markers, and pinpoint the risk factors contributing to LE8. Ultimately, this investigation lays a theoretical foundation for the long-term cardiovascular health management and targeted interventions for RA and LSE patients.

## Objects and methods

### Objects

The method of cross-sectional study was used to select adult rheumatic patients who were treated in the general clinic and inpatient department of the Second Hospital of Shanxi Medical University from May 2022 to May 2023. Inclusion criteria are as follows: (1) the diagnostic criteria for patients with systemic lupus erythematosus refer to the SLE classification standards established by EULAR/ACR (European League Against Rheumatism/American College of Rheumatology, EULAR/ACR) in 2019 [10] and the diagnosis of RA patients conforms to the diagnostic and classification criteria of RA specified by EULAR/ACR version 2010 [11]; (2) age ≥ 18 years old; (3) clear consciousness and normal communication, volunteer to participate in this study; (4) those with complete data of 8 ideal cardiovascular behaviors and factors in the baseline data. Exclusion criteria are as follows: (1) pregnant women, (2) patients with severe organ diseases, (3) acute trauma and infection, (4) patients with malignant tumor.

### Methods

**Fig. 1:**
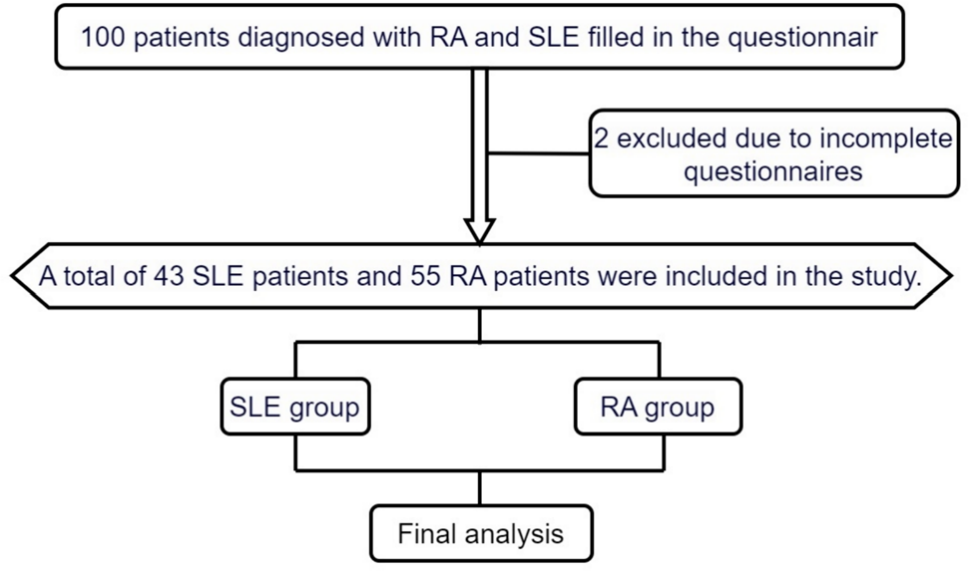
Research flowchart

1. Questionnaire survey: A questionnaire is used to collect the general situation of the population, past history, medication history, etc., which are asked or filled out by specially trained medical staff. The contents of the survey include the following: sex, age, place of residence, income, smoking, living habits, physical activity, chronic medical history (hypertension, diabetes, coronary heart disease, and other cardiovascular disease history and medication), and disease activity, course of disease (calculated from the time of the first definite diagnosis by the doctor); the diet was slightly adjusted in this study. According to the Dietary Guidelines for Chinese residents (2022), the dietary intake was divided into eight items: whole grains, vegetables, fruits, aquatic products, lean meat and eggs, milk, salt, oil, and sugar (Table 1).
2. Anthropometry: this includes height, body mass, waist circumference, blood pressure, and BMI (body mass index, BMI). Measurement of height includes barefoot without crown, coat off, arms drooping naturally, heel close, 45 between feet, feet, buttocks, shoulders and back brain close to height ruler and stand upright, eyes usually front, height reading accurate to 0.1 cm, weight reading accurate to 0.1 kg.
3. Laboratory examination: 10 mL was drawn from the venous blood of the subjects in the early morning and fasting for 8 to 12 h. C-reactive protein (CRP), erythrocyte sedimentation rate (ESR), fasting blood glucose (FBG), glycated hemoglobin glycosylated hemoglobin (HbA1c), total serum cholesterol (TC), serum triglyceride (TG), and non-high-density lipoprotein (nHDL-C) were measured by the automatic biochemical analyzer. The indexes of blood lipids were determined by enzymatic method, all of which were tested by the unified medical laboratory of the country.
4. The definition of LE8:LE8 includes four health behaviors: diet, physical activity, nicotine exposure, BMI, and 4 health factors: blood sugar and glycosylated hemoglobin, blood pressure, blood lipids, and sleep health. According to the definition of LE8, the above eight items were assigned according to the criteria, with a range of 0 ~ 100. The total LE8 score was defined as the unweighted average of the 8 items, with 0 ~ 49 as low, 50 ~ 79 as medium, and 80 ~ 100 as high (Table 1).
5. Disease activity assessment: SLEDAI (Systemic Lupus Erythematosus Disease Activity Index, SLEDAI) was used to evaluate the disease activity of SLE patients, including 24 clinical symptoms, with a total score of 105,in the disease activity assessment, 4 as basically inactive, 5 to 9 as mild activity, 10 to 14 as moderate activity, and ≥ 15 as severe activity. The Disease Activity Score derivative for 28 joints (DAS28) was used to evaluate the disease activity of RA patients. The DAS28 score was calculated based on the number of swollen joints, tenderness joints, ESR, and CRP. The DAS28 score was defined as remission, 2.6 to 3.2, 3.3 to 5.1, and high (Fig. 1).
6. Cardiovascular disease risk prediction: This study uses the Prediction for ASCVDR Risk in China (China⁃PAR) model to evaluate the 10-year and lifetime cardiovascular risks of the study subjects. The 10-year risk of cardiovascular disease ≥ 10.0% is considered a high risk of cardiovascular disease. 5.0 to 9.9% is considered medium risk, and < 5.0% is considered low risk. Lifetime cardiovascular risk assessment was performed on patients aged 20 to 59 years. A lifetime risk < 32.8% was considered a lifetime low risk, and a lifetime risk ≥ 32.8% was considered a lifetime high risk.

**Table 1:** The definition of LE8

LS8 metric	Measurement methods	Scoring criteria
Diet score	Via questionnaire	Metric: number of healthy eating items Scoring: Points, level 100, 7 ~ 8 80, 5 ~ 6 50, 3 ~ 4 25, 1 ~ 2 0, 0
Physical activities score	Self-reported minutes of moderate- or vigorous-intensity PA per week	Metrics: minutes of moderate-intensity (or higher-intensity) activity per week Scoring: Points, minutes 100, ≥ 150 90, 120 ~ 149 80, 90 ~ 119 60, 60 ~ 89 40, 30 ~ 59 20, 1 ~ 29 0, 0
Smoking score	Measurements: self-reported smoking or use of inhaled nicotine delivery system NDS	Metric: combustible tobacco use or inhaled NDS use; or secondhand smoke exposure Scoring: Points, status 100, never 75, quit smoking ≥ 5 years 50, 1 ≤ quit smoking < 5 years 25, quit smoking for < 1 year, or currently using an inhaled NDS (nicotine delivery system, such as e-cigarettes) 0, current smoker 20, points deducted for living in a home with an active indoor smoker (unless it is 0 points)
Sleep score	Measurement: self-reported average sleep duration per night	Metric: Average hours of sleep per night: Scoring: Points, level 100, 7 ~ < 9 90, 9 ~ < 10 70, 6 ~ < 7 40, 5 ~ < 6 or ≥ 10 20, 4 ~ < 5 0, < 4
BMI score	Measurement method: measure weight (kg) divided by height squared (m^2^)	Metric: BMI (kg/m^2^) Scoring: Points, level 100, < 25 70, 25.0 ~ 29.9 30, 30.0 ~ 34.9 15, 35.0 ~ 39.9 0, ≥ 40.0
Blood pressure score	Measure systolic and diastolic blood pressure	Metric: systolic and diastolic BPs (mm Hg) Scoring: Points, level 100, < 120/ < 80 75, 120 ~ 129/ < 80 50, 130 ~ 139 or 80 ~ 89 25, 140 ~ 159 or 90 ~ 99 0, ≥ 160 or ≥ 100 If the drug-treated level, subtract 20 points
Lipid score	Measurements: plasma total cholesterol and HDL cholesterol, calculated non-HDL cholesterol	Indicator: non-HDL cholesterol (mmol/L) Scoring: Points, standard (mmol/L) 100, < 3.36 60, 3.36 ~ 4.10 40, 4.11 ~ 4.88 20, 4.89 ~ 5.66 0, ≥ 5 0.67 If medication is at the therapeutic level, deduct 20 points
Blood sugar score	Measurement: fasting (FBG, HbA1c) or non-fasting (HbA1c) blood samples	Metric: FBG (mg/dL) or HbA1c (%) Scoring: Points, level 100, no diabetes and FBG < 5.55 (or HbA1c < 5.7) 60, no history of diabetes and FBG: 5.56 to 6.95 (or HbA1c 5.7 to 6.4) (prediabetes) 40, having diabetes with HbA1c < 7.0 30, having diabetes with HbA1c 7.0 to 7.9 20, having diabetes with HbA1c 8.0 to 8.9 10, having diabetes with HbA1c 9.0 to 9.9 0, having diabetes with HbA1c ≥ 10.0

### Statistical method

After verifying the data, use SPSS 26.0 software to establish a database and input the data and analyze the relevant data. The main research factors are assigned values; see Table 1. The Shapiro–Wilk test is used for normal distribution testing. If the measurement data conforms to the normal distribution, it is expressed by( $\overline{x }$±s), and the *t* test is used for comparison between groups; if the measurement data does not conform to the normal distribution, it is expressed by the median (interquartile range), and the rank sum test is used for comparison between groups; count data comparisons between groups were performed using the *χ*2 test. When the group frequency was 0, Fisher’s precision probability test was used. Data correlation was performed using Spearman Correlation Analysis or Pearson Correlation Analysis.). Variables with *P* < 0.05 were selected from the single factor analysis and entered into the logistic regression model for risk factor analysis. *P* < 0.05 means the difference is statistically significant. The enumeration data are expressed as the number of cases (%), and the test level is *α* = 0.05. *P* < 0.05 is considered to be statistically significant. When a variable is missing in the study, the method of column deletion is adopted during statistical analysis. The analysis is conducted after deletion.

## Result

### Baseline general characteristics of study subjects and distribution of cardiovascular health scores

#### General situation of the studied population and LE8 grading

A total of 98 patients with rheumatism, 43 patients with SLE, and 55 patients with RA were included. Finally, 98 patients with valid data were included, including 11 males and 87 females, with an average age of 49.12 ± 15.86 years old. There were 10 (18.18%), 36 (65.45), and 9 (16.36%) cases in the RA group with high, medium, and low LE8 scores respectively. Among the eight-item scores, exercise score was the lowest (33.27 ± 45.46), followed by diet (44.18 ± 18.80). In the SLE group, there were 13 cases (30.23%), 30 cases (69.77%), and 0 cases (0%) with low LE8 scores, respectively. Among the eight-item scores, the exercise score was the lowest (38.14 ± 44.36), and the diet score was the lowest (50.81 ± 23.50). In both groups, most met the moderate LE8 level, and none met the full LE8. See Table 2 for details.

**Table 2:** Baseline comparison of RA and SLE patient groups

Project	Overall (*n* = 98)	RA group (*n* = 55)	SLE group (*n* = 43)	*P*
Age (years)	49.12 ± 15.86	58.65 ± 10.22	36.64 ± 13.04	< 0.01
Gender (number of cases)				
Male	11 (11.22%)	6 (10.91%)	5 (11.63%)	0.911
Female	87 (88.78%)	49 (89.09%)	38 (88.37%)	
BMI (Kg/m^2^)	23.06 (20.51, 25.98)	23.29 ± 3.61	22.82 (19.51, 27.09)	0.054
Systolic blood pressure (mmHg)	122 (11.5, 132.5)	127.89 ± 15.27	120.63 ± 13.85	0.017
Diastolic blood pressure (mmHg)	76.60 ± 11.23	78.32 ± 9.63	73 (63.00, 81.00)	0.085
TC (mmol/L)	4.47 (3.96, 5.21)	4.65 ± 1.12	4.26 (3.92, 5.15)	0.705
TG (mmol/L)	1.40 (1.03, 2.02)	1.46 (0.95, 2.25)	1.56 ± 0.62	0.445
nHDL-C (mmol/L)	3.31 (1.98, 3.92)	3.41 (2.06, 3.97)	3.03 (1.88, 3.92)	0.480
FBG (mmol/L)	4.96 (4.34, 5.55)	5.26 (4.52, 5.73)	4.73 ± 0.64	0.002
Education level (number of cases)				
High school or above	48 (48.98%)	19 (34.55%)	29(67.44%)	0.002
Below high school	50 (51.02%)	36 (65.45%)	14 (32.56%)	
Annual household income per capita (number of cases)				
30,000 or above	28 (28.57%)	15 (27.27%)	13 (30.23%)	0.075
Below 30,000	70 (71.43%)	40 (72.73%)	30 (69.77%)	
LE8 score	68.82 ± 12.29	64.48 ± 12.26	74.36 ± 9.9	< 0.01
Factor score	77.05 ± 14.30	72.46 ± 11.99	83.34 ± 14.90	< 0.01
Behavior score	60.91 ± 17.78	56.50 ± 18.81	66.22 ± 14.75	< 0.01
80–100	23 (23.47%)	10 (18.18%)	13 (30.23%)	0.148
50–79	66 (67.35%)	36 (60.00%)	30 (67.99%)	
0–49	9 (9.18%)	9 (16.36%)	0 ( 0.00%)	

Note: The *P*-value represents the comparison between the SLE group and the RA group. The asterisk (*) indicates *P* < 0.05

#### LE8 scores of patients with RA and SLE

Among the study population, 23 cases (23.67%) were highly satisfied with ideal cardiovascular health, 2 were male (8.70%), and 21 were female (91.30%). Fifty-six cases (57.14%) were moderately satisfied with ideal cardiovascular health, and 7 were male (12.50%). %), 49 cases were female (87.5%), 9 cases met low ideal cardiovascular health (9.18%), 2 cases were male (22.22%), and 7 cases were female (77.78%). The diet score was the lowest in the high LE8 group, and the exercise score was the lowest in the medium and low LE8 groups (Table 3).

**Table 3:** Distribution of ideal cardiovascular health behaviors and factors

Project	High LE8 group (*n* = 23)	Medium LE8 group (*n* = 56)	Low LE8 group (*n* = 9)
Age (year)	42.96 ± 16.85	49.73 ± 15.41	60.89 ± 10.39
Gender (number of cases)			
Male	2 (8.70%)	7 (12.50%)	2 (22.22%)
Female	21 (91.30%)	49 (87.50%)	7 (77.78%)
BMI score	100 (100, 100)	100 (75, 100)	100 (85, 100)
Blood pressure score	75 (75, 100)	75 (50, 100)	36.11 ± 22.05
Blood sugar score	100 (87, 100)	78.17 (60, 100)	60 (40, 80)
Lipid score	90 (82.5, 100)	67.86 (60, 100)	60 (32.5, 80)
Smoking score	100 (100, 100)	100 (100, 100)	100 (5, 100)
Physical activities score	100 (90, 100)	0 (0, 20)	0 (0, 0)
Diet score	50 (50, 80)	50 (25, 50)	25 (25, 37.5)
Sleep score	100 (70, 100)	70 (40, 100)	40 (20, 40)
Education level (number of cases)			
High school or above	14 (60.87%)	21 (37.50%)	2 (22.22%)
Below high school	9 (39.13%)	35 (62.50%)	7 (77.78%)
Annual household income per capita (number of cases)			
30,000 or above	10 (43.48%)	7 (12.50%)	1 (11.11%)
Below 30,000	13 (56.52%)	49 (87.50%)	8 (88.89%)
LE8 score	84.74 ± 4.64	66.11 ± 7.53	47.65 ± 2.64
LE8 factor score	87.58 ± 9.102	76.21 ± 13.98	61.69 ± 7.82
LE8 behavior score	82.17 ± 8.77	56.45 ± 11.94	33.61 ± 8.73

The score for LE8 in the study population was 68.78 ± 12.32, with a behavioral score of 59.90 ± 18.57 and a factor score of 77.63 ± 14.77. The total LE8 score for the SLE group was 74.36 ± 9.9, with a behavioral score of 66.22 ± 14.75 and a factor score of 83.34 ± 14.90. The total LE8 score for the RA group was 64.48 ± 12.26, with a behavioral score of 56.50 ± 18.81 and a factor score of 72.46 ± 11.99.

### Factors influencing LE8 scores in SLE patients

#### LE8 scores tend to be higher among SLE patients who are young, have high incomes, or have low disease activity

The *χ*^2^ test was employed to investigate the association between various factors such as gender, age, economic status, educational level, comorbidity with cardiovascular disease, SLEDAI (Systemic Lupus Erythematosus Disease Activity Index) scores, duration of illness, and LE8 scores in patients with SLE. Among the SLE cohort, those with an annual household income exceeding 30,000 per capita (*P* = 0.027), those aged 35 years or below (*P* = 0.007), and those with lower SLEDAI scores (*P* = 0.00) demonstrated significant associations with LE8 scores. Among the factors considered, gender, age, education level, and the presence of other cardiovascular diseases did not exhibit statistical significance in the LE8 group (*P* > 0.05). For further details, please refer to Table 4.

**Table 4:** Analysis of the relationship between LE8 risk factors in SLE group

	Medium LE8 group	High LE8 group	*χ*^2^/*H* (K)	*P*
Age (number of cases)			7.615	0.007**
> 35 years	40 (66.67%)	15 (38.46%)		
≤ 35 years	20 (33.33%)	24 (61.54%)		
Gender (number of cases)			0.762	0.519
Male	26 (86.67%)	12 (92.30%)		
Female	4 (13.33%)	1 (7.70%)		
Annual household income per capita (number of cases)			5.627	0.027*
30,000 or above	46 (76.67%)	21 (53.85%)		
Below 30,000	14 (23.33%)	18 (46.15%)		
Education level (number of cases)			2.030	0.187
High school or above	22 (36.67%)	9 (23.08%)		
Below high school	38 (63.33%)	30 (76.92%)		
Combined cardiovascular disease (number of cases)			0.937	0.380
Yes	2 (3.33%)	3 (7.69%)		
No	58 (96.67%)	36 (92.31%)		
SLEDAI Score Classification (number of cases)			22.416	< 0.01**
0–4	1	9		
5–9	8	1		
10–14	12	1		
≥ 15	3	0		
Course of disease	8 (3, 17)	4 (2.5, 15)	0.273	0.601

Note: The asterisk (*) indicates *P* < 0.05, while the asterisks (**) indicate *P* < 0.01

#### Multivariate logistic regression analysis: high disease activity is an independent risk factor for LE8 scores in SLE patients

We evaluated and collected SLEDAI indices from 35 LSE patients, and after excluding confounding variables such as gender, disease duration, and educational attainment, we conducted an ordered multicategory logistic regression analysis. Our findings indicated that factors such as age ≤ 35 years, family per capita annual income exceeding 30,000, and SLEDAI score did not exhibit statistical significance in predicting a one-scale increase in LE8 (*P* > 0.05). The established Model 1 was statistically significant (*χ*^2^ = 21.578, *P* < 0.01), indicating its validity and reliability in elucidating the relationships among the studied variables. After adjusting for confounding factors including gender, disease duration, educational level, age, and annual family income per capita, an ordered multi-category logistic regression analysis was conducted to investigate the association between the SLEDAI score and LE8 score among patients in the SLE group; the analysis revealed that for every increase in the SLEDAI score, the probability of an upward grade shift in the LE8 score is 0.07 times (95% CI: 0.01 ~ 0.37, *P* < 0.05). The established model 2 is statistically significant (*χ*^2^ = 21.150, *P* ≤ 0.01). See Table 5.

**Table 5:** Multivariate analysis of risk factors for LE8 in the SLE group

	Factor	*β*	Warld χ2	*P*	OR (95% CI)
Model 1	SLEDAI score classification	− 2.520	8.564	0.003**	0.08 (0.00 ~ 0.35)
	Age (≤ 35 years = 1, > 35 years = 2)	0.742	0.232	0.630	2.10 (0.10 ~ 42.82)
	Annual household income per capita (≤ 30,000 = 1, > 30,000 = 2)	− 1.096	0.394	0.530	0.10 (0.33 ~ 9.36)
Model 2	SLEDAI score classification	− 2.647	9.733	0.002**	0.07 (0.01 ~ 0.37)

Note: Model 1 corrects the confounding factors of gender, educational level, and comorbid diseases. Model 2 corrects the confounding factors of gender, educational level, comorbid diseases, age, and per capita annual household income. The asterisk (*) indicates *P* < 0.05, while the asterisks (**) indicate *P* < 0.01

The Spearman correlation analysis was employed to assess the relationship between the SLEDAI score and the LE8 score. The results indicated a negative correlation between the LE8 score and both the ideal cardiovascular factor score and the ideal cardiovascular behavior score among SLE patients, with correlation coefficients of *r* =  − 0.807, − 0.556, and − 0.527, respectively (*P* < 0.05).

#### Relationship between inflammatory indicators, complement levels, anti-ds-DNA levels, and LE8 in SLE patients

We collected and analyzed the levels of ESR (38 cases), CRP (36 cases), C3 complement (35 cases), C4 (35 cases), and anti-ds-DNA (29 cases) indicators in SLE patients. Upon analyzing the associations between CRP (*P* = 0.082), ESR (*P* = 0.366), C3 (*P* = 0.130), C4 (*P* = 0.317), and anti-ds-DNA antibody levels (*P* = 0.276) with the LE8 score, LE8 behavioral score, and LE8 factor score in SLE patients, we discovered that none of these markers exhibited a statistically significant correlation with the LE8 score. In the SLE cohort, there was no statistically significant correlation between CRP (*P* = 0.994), ESR (*P* = 0.453), C4 (*P* = 0.298), and anti-ds-DNA antibody levels (*P* = 0.712) with the LE8 behavioral score. However, a moderate negative correlation was observed between C3 levels and the LE8 behavioral score (*r* =  − 0.384, *P* = 0.023).

### Factors influencing LE8 scores in RA patients

#### The impact of age, gender, income, duration of illness, and disease activity on LE8 grading in RA patients

We evaluated and collected the DAS28 levels of 40 RA patients and conducted an analysis. The *χ*^2^ test or rank sum test was used to analyze the relationship between gender, age, economic level, educational level, DAS28 score, disease course, and LE8 score in the RA group. Among patients with RA, there were no statistically significant differences in LE8 scores among different genders, ages, economic levels, educational levels, and disease durations (*P* > 0.05). After grouping according to the DAS28 score, Fisher’s exact test was used to analyze the LE8 distribution in different DAS28 groups. It was found that there were differences in LE8 distribution between the groups. The lower the DAS28 score, the higher the proportion of high LE8 scores (*P* < 0.01). See Table 6.

**Table 6:** Analysis of LE8 risk factors in the RA group

Factor	Low LE8 group	Medium LE8 group	High LE8 group	χ^2^/*H* (K)	*P*
Age (number of cases)				2.977	0.183
> 50 years	9 (100.00%)	29 (80.56%)	7 (70.00%)		
≤ 50 years	0 (0.00%)	7 (19.44%)	3 (30.00%)		
Gender (number of cases)				1.016	0.706
Male	7 (77.78%)	23 (88.46%)	9 (90.00%)		
Female	2 (22.22%)	3 (11.54%)	1 (10.00%)		
Annual household income per capita (number of cases)				2.006	0.381
30,000 or above	8 (88.89%)	26 (72.22%)	6 (60.00%)		
Below 30,000	1 (11.11%)	10 (27.78%)	4 (40.00%)		
Education level (number of cases)				0.775	0.771
High school or above	7 (77.78%)	23 (88.46%)	6 (60.00%)		
Below high school	2 (22.22%)	13 (11.54%)	4 (40.00%)		
DAS28 score classification (number of cases)				76.11	< 0.01
< 2.6	0	1	10		
2.7 ~ 3.2	0	14	0		
3.3 ~ 5.1	0	15	0		
> 5.1	6	6	0		
Course of disease	13 (6.5, 20)	7.5 (3, 12.5)	7 (6, 15)	2.4.57	0.293

Note: the asterisk (*) indicates *P* < 0.05; the asterisks (**) indicate *P* < 0.01

#### Relationship between disease activity and LE8 in RA patients

Spearman correlation was used to analyze the correlation between the DAS28 score and the LE8 score. The LE8 score, ideal cardiovascular factor score, and ideal cardiovascular behavior score of RA patients were negatively correlated with the DAS28 score, and the correlation coefficients were − 0.96, − 0.646, and − 0.789, respectively (*P* < 0.05). Ordered multi-factor logistic regression analysis was conducted to investigate the association between LE8 grading and disease course, as well as disease activity grading in the RA cohort. The results revealed that neither disease duration nor DAS28 grading exhibited a statistically significant influence on LE8 grading (*P* > 0.05).

#### Relationship between inflammatory markers, antibodies, and LE8 in RA patients

We gathered and examined the EAR and CRP inflammation index levels from 47 RA patients for analysis. The analysis revealed no statistically significant correlation between CRP and LE8 score (*P* = 0.765), LE8 behavior score (*P* = 0.931), or LE8 factor score (*P* = 0.609) in patients with RA. Similarly, no significant correlation was observed between ESR and LE8 score (*P* = 0.575), LE8 behavior score (*P* = 0.430), or LE8 factor score (*P* = 0.258). We conducted further analysis to explore the relationship between rheumatoid factor (RF), anti-cyclic peptide-containing citrulline (anti-CCP), and LE8. For this, we gathered data on RF and anti-CCP from 38 RA patients and examined the correlation between these antibodies and LE8 scores. However, our findings revealed no statistically significant correlation.

### Individuals with high LE8 scores have a lower 10-year cardiovascular risk

Using the China-PAR model, the 10-year cardiovascular risk was 6.46 ± 7.01% and the lifetime cardiovascular risk was 27.09 ± 10.91% in the RA group. In the SLE group, the 10-year cardiovascular risk was 3.04 ± 3.73%, and the lifetime risk was 22.51 ± 10.59%. Spearman correlation was used to analyze the correlation between 10-year risk and lifetime risk and LE8 score. The results showed a moderate negative correlation, *r* =  − 0.49 (*P* < 0.01), − 0.580 (*P* < 0.01).

The population was divided into the low-risk group (< 5.0%), the intermediate-risk group (5.0 ~ 9.9%), and the high-risk group (≥ 10%) based on the 10-year cardiovascular risk stratification estimated by the China-PAR model. In the RA patient group, there were 5 cases (9.10%), 23 cases (41.80%), and 27 cases (49.10%) in the low-risk, intermediate-risk, and high-risk groups, respectively; in the SLE group, based on the 10-year cardiovascular risk, there were 19 cases (44.20%), 19 cases (44.20%), and 5 cases (11.60%) in the low-risk group, the intermediate-risk group, and the high-risk group, respectively. Ordinal logistic regression was used to analyze the relationship between 10-year cardiovascular risk and LE8 scores. As the LE8 score increases, the risk of increased cardiovascular risk stratification in 10 years decreases, with an OR value of 0.45 (95% CI, 0.23–0.88; *P* = 0.002). Single-factor logistic order was used to analyze the relationship between LE8 grades of different diseases and 10-year cardiovascular risk. It was found that for RA patients whose LE8 increased by one grade, the 10-year cardiovascular risk stratification risk increased by 0.03 times the original value (95% CI, 0.00 ~ 0.22; *P* = 0.001); for SLE patients, the 10-year cardiovascular risk stratification risk increased by one degree for each increase in LE8 was 0.06 times the original (95% CI, 0.01 ~ 0.35; *P* = 0.002).

A total of 65 patients aged 20 to 59 years were included in the population, 29 in the RA group, and 36 in the SLE group. According to the lifetime cardiovascular risk stratification evaluated by the China-PAR model, they were divided into the low-risk group (< 32.8%) and the high-risk group (≥ 32.8%). In the RA patient group, there were 6 cases (20.70%) and 23 cases (79.30%) in the low-risk group and high-risk group, respectively; in the SLE group, there were 22 cases (61.10%) in the low-risk group and 14 cases (38.90%) high-risk group, respectively. Ordered logistic regression was used to analyze the relationship between lifetime cardiovascular risk and LE8 scores. There was no difference in lifetime cardiovascular risk stratification between different LE8 scores. Single-factor logistic order was used to analyze the relationship between LE8 grading of different diseases and lifetime cardiovascular risk. It was found that there was no difference in lifetime cardiovascular risk scores between different LE8 grading groups in the RA group and SLE group (*P* > 0.05).

## Discussion

This comprehensive study utilized the LE8 model to evaluate the cardiovascular health of RA and SLE patients, while also delving into the risk factors that influence LE8 levels. Notably, the research revealed a strong correlation between age, economic level, and disease activity with LE8, particularly highlighting high disease activity as an independent risk factor for reduced LE8 in SLE patients. Furthermore, the study found that the LE8 level mirrors the 10-year cardiovascular risk, indicating that attaining an optimal LE8 level through targeted interventions could serve as an effective strategy to mitigate cardiovascular risk.

The risk of cardiovascular disease in RA patients is 3.96 times that of the general population [12], and the risk of concurrent CVD is 48% higher than that of the general population [13]. The prevalence of CVD among SLE patients can reach 9.7%, and the risk of coronary heart disease can reach 7 to 8 times that of the general population [14]. Cardiovascular health issues should be considered in the long-term management of patients with rheumatic diseases.

Following the introduction of the ICH concept in 2020, subsequent research has established that a higher ICH score serves as a deterrent to rheumatic diseases. A foreign study examining the correlation between ICH and the risk of RA revealed that among the 17,532 individuals studied, men over 50 years old in the 4th quantile of ICH had a risk of RA that was 0.260 times lower than those in the 1st quantile. Similarly, for women in the same age group, the risk of RA in the 4th quantile was 0.313 times lower compared to those in the 1st quantile [8]. It is suggested that there is a close correlation between the ICH score and the risk of RA, where a higher ICH score corresponds to a lower risk of developing RA. The study conducted by Wang et al. [15] concurs with this finding. This indicates that by promoting early popularization and achieving LE8 targets among the population, there is potential to significantly decrease the incidence rate of RA in patients. The introduction of the ICH concept not only presents a novel approach for preventing rheumatic diseases but also furnishes valuable suggestions for managing cardiovascular health in individuals afflicted with rheumatic illnesses. In a foreign study examining the ICH status of 76 female SLE patients, brachial-ankle pulse wave velocity (baPWV) served as a biological marker for assessing arteriosclerosis severity. Recognized as an independent risk factor for arteriosclerosis, baPWV indicates mild sclerosis when ranging from 1400 to 1800 cm/s and suggests the presence of peripheral arteriosclerosis when exceeding 1800 cm/s. The study revealed that female SLE patients who fulfilled 3 to 4 ICH criteria exhibited a baPWV that was 110 cm/s lower compared to those who only met 0 to 2 ICH criteria. Furthermore, a direct correlation was observed between the number of ICH criteria met and a decrease in the arteriosclerosis index, as reported in [16]. A study conducted in the USA in 2024 revealed that RA patients boasting high LE8 scores exhibited a reduced risk of all-cause mortality. Specifically, for each 1-point elevation in LE8, the mortality risk among these patients diminished by 2.6%. Nevertheless, additional exploration into the correlation between LE8 and disease activity remains absent at present.

For the first time, this study employed the LE8 model to evaluate the cardiovascular health of individuals suffering from RA and SLE. The analysis encompassed the distribution of these conditions and the identification of risk factors influencing the LE8 distribution. In 2023, a comprehensive study was conducted in China to investigate the distribution of LE8 among residents. The study employed stratified sampling, drawing from 289 counties across 31 provinces, and encompassed 70,093 individuals aged 20 and above. Upon analysis, it was discovered that the mean LE8 score for this demographic stood at 73.3 ± 12.6. Notably, females exhibited a higher LE8 score (77.9 ± 11.6) compared to their male counterparts (68.7 ± 11.8). Furthermore, the study found that 33.0% of residents had higher LE8 scores, 63.2% fell into the medium category, and 3.9% had lower scores [17]. In this study, the subjects’ average LE8 score stood at 68.82 ± 12.29. Specifically, the SLE group secured a score of 74.36 ± 9.9, whereas the RA group obtained a score of 64.48 ± 12.26. It was observed that the LE8 level of the RA group fell below the average LE8 level among Chinese residents, whereas the SLE group’s overall LE8 level matched the national average. Previous studies have demonstrated that individuals who are younger, female, and have a higher level of educational attainment tend to have higher LE8 scores [17]. In our study, the mean age of patients in the SLE group (36.64 ± 13.04) was notably lower compared to the RA group (58.65 ± 10.22), and their educational background was more advanced. These factors could potentially explain why the SLE group exhibited a higher LE8 score than the RA group in our research. Additionally, it was discovered that the LE8 behavior scores were inferior to the factor scores in both groups. Among the eight indicators, physical exercise fared the worst, closely followed by diet. This observation could be attributed to the fact that RA is an inflammatory joint disease that limits physical movement. Moreover, SLE patients frequently encounter multiple system involvement, which may lead to joint pathologies, ultimately impeding physical activities. Therefore, greater emphasis should be placed on the cardiovascular health of rheumatic patients, and efforts to strengthen interventions aimed at improving their cardiovascular well-being are essential. Specifically, within lifestyle interventions, the primary focus should be on actively managing the underlying disease while prioritizing the promotion of physical exercise and a balanced diet.

Previous studies have established a correlation between factors like smoking, physical activity, hypertension, and hyperglycemia within the LE8 framework and an elevated cardiovascular risk among rheumatic patients [18–20]. Separate investigations have revealed that, even after accounting for conventional risk factors including age, smoking, and dyslipidemia, the activity of RA and SLE diseases continues to elevate cardiovascular risk, with this linkage being particularly pronounced during the early phases of the illnesses [21–24]. Studies have shown that approximately 30% of cardiovascular events in RA patients can be traced back to variations in disease activity, inflammatory markers, or changes in specific antibody levels [25]. Through the utilization of the LE8 score, our study provides additional evidence that LE8 is closely linked to disease activity in rheumatic patients. Specifically, we observed that as the SLEDAI or DAS28 scores escalate, the LE8 score progressively diminishes. Hence, early treatment and intervention targeted at diminishing disease activity in rheumatic patients can significantly enhance their cardiovascular well-being. Research has demonstrated that an escalation in inflammatory markers heightens the cardiovascular risk for these patients. This correlation might stem from the impact of elevated CRP and ESR levels on lipid regulation and endothelial function in individuals with RA and SLE, factors that contribute to the onset of cardiovascular ailments [26–28]. In our study, the aforementioned relationship was not observed. When further exploring the relationship between related antibodies and LE8, it was found that there was no significant correlation between RF, anti-CCP antibodies, and LE8, which could be attributed to the small sample size, and this is something we need to improve in our next study. However, our research results revealed a negative correlation between anti-ds-DNA levels in SLE patients and LE8 factor scores. This correlation is attributed to the fact that consistently elevated anti-ds-DNA antibodies can induce endothelial dysfunction and facilitate the development of atherosclerosis, as supported by previous research [29]. It is evident that aggressive management of the underlying disease and substantial reduction in disease activity plays a pivotal role in mitigating cardiovascular risk. This approach not only aids in decreasing the incidence of cardiovascular events but also enhances the overall cardiovascular well-being significantly. Hence, effective control and administration of the primary ailment, coupled with diminishing disease activity, constitute a vital health preservation step.

Currently, there is no widely accepted cardiovascular risk prediction model specifically tailored for patients with rheumatic diseases. Traditional cardiovascular risk models, such as the Framingham and Systematic Coronary Risk Evaluation (SCORE) systems, frequently underestimate the cardiovascular risk associated with rheumatic diseases. Therefore, the EULAR recommends the use of a multiplication factor of 1.5 to enhance cardiovascular risk assessment in this patient population [30]. This study employs the China-PAR model, specifically tailored to assess cardiovascular risk among Chinese individuals, to assess the 10-year risk for RA and SLE patients, applying a multiplicative factor of 1.5 for evaluation purposes. After examining the correlation between the China-PAR model and LE8, it was discovered that the RA group exhibited a moderate 10-year cardiovascular risk of 7.43 ± 3.76%, while the SLE group demonstrated a lower risk of 4.55 ± 5.59%. Notably, the RA cohort was classified as having a moderate 10-year cardiovascular risk, whereas the SLE group exhibited a lower risk profile. This disparity could potentially be explained by the comparatively younger mean age of the SLE patients. In this study, after analyzing the correlation between 10-year cardiovascular risk and LE8, we discovered that a higher LE8 level acts as a safeguarding element for 10-year cardiovascular risk. Furthermore, across various disease cohorts, it was observed that an increase in the LE8 score corresponded to a decrease in the likelihood of a higher China-PAR risk stratification. This finding provides additional evidence supporting the protective role of LE8 in maintaining the cardiovascular well-being of individuals afflicted with rheumatic illnesses.

## Advantages and limitations

Currently, foreign studies have established a link between LE8 and the risk of RA. However, they have yet to delve into its association with cardiovascular risk and disease activity among rheumatism patients. This study represents the initial endeavor to utilize the LE8 model in assessing the cardiovascular health status of rheumatism patients. Additionally, it aims to investigate the correlation between LE8 and disease activity as well as relevant inflammatory markers among patients with rheumatic diseases. Nonetheless, this study is not without limitations. Specifically, the sample size employed in this investigation is relatively small. Consequently, the subsequent phase involves expanding the sample size and incorporating cytokines and autoantibodies for a more comprehensive analysis.

## Supplementary Information

Below is the link to the electronic supplementary material.

Supplementary file1 (DOC 84 KB)
